# Lipidated Dengue-2 Envelope Protein Domain III Independently Stimulates Long-Lasting Neutralizing Antibodies and Reduces the Risk of Antibody-Dependent Enhancement

**DOI:** 10.1371/journal.pntd.0002432

**Published:** 2013-09-19

**Authors:** Chen-Yi Chiang, Chien-Hsiung Pan, Chun-Hsiang Hsieh, Jy-Ping Tsai, Mei-Yu Chen, Hsueh-Hung Liu, Shih-Jen Liu, Pele Chong, Chih-Hsiang Leng, Hsin-Wei Chen

**Affiliations:** 1 National Institute of Infectious Diseases and Vaccinology, National Health Research Institutes, Miaoli, Taiwan, Republic of China; 2 Graduate Institute of Immunology, China Medical University, Taichung, Taiwan, Republic of China; Florida Gulf Coast University, United States of America

## Abstract

**Background:**

Dengue virus is a mosquito-transmitted virus that can cause self-limiting dengue fever, severe life-threatening dengue hemorrhagic fever and dengue shock syndrome. The existence of four serotypes of dengue virus has complicated the development of an effective and safe dengue vaccine. Recently, a clinical phase 2b trial of Sanofi Pasteur's CYD tetravalent dengue vaccine revealed that the vaccine did not confer full protection against dengue-2 virus. New approaches to dengue vaccine development are urgently needed. Our approach represents a promising method of dengue vaccine development and may even complement the deficiencies of the CYD tetravalent dengue vaccine.

**Methodology/Principal Findings:**

Two important components of a vaccine, the immunogen and immunopotentiator, were combined into a single construct to generate a new generation of vaccines. We selected dengue-2 envelope protein domain III (D2ED III) as the immunogen and expressed this protein in lipidated form in *Escherichia coli*, yielding an immunogen with intrinsic immunopotentiation activity. The formulation containing lipidated D2ED III (LD2ED III) in the absence of exogenous adjuvant elicited higher D2ED III-specific antibody responses than those obtained from its nonlipidated counterpart, D2ED III, and dengue-2 virus. In addition, the avidity and neutralizing capacity of the antibodies induced by LD2ED III were higher than those elicited by D2ED III and dengue-2 virus. Importantly, we showed that after lipidation, the subunit candidate LD2ED III exhibited increased immunogenicity while reducing the potential risk of antibody-dependent enhancement of infection in mice.

**Conclusions/Significance:**

Our study suggests that the lipidated subunit vaccine approach could be applied to other serotypes of dengue virus and other pathogens.

## Author Summary

Vaccines are considered a cost-effective way to control infectious diseases. To rationally design vaccines, antigens and, frequently, adjuvants must be selected to trigger appropriate immune responses against a specific pathogen. We selected dengue-2 envelope protein domain III as a dengue vaccine candidate and expressed this candidate in the lipidated form in an *Escherichia coli*-based system. Dengue envelope protein domain III mediates binding of the dengue virus to the host cellular receptor. The lipid moiety of the bacterial-derived lipoprotein can activate the innate immune system to elicit an appropriate adaptive immune response. We demonstrated that lipidated dengue-2 envelope protein domain III is more immunogenic than nonlipidated dengue-2 envelope protein domain III. Most importantly, the lipidated dengue-2 envelope protein domain III alone triggered a durable neutralizing antibody response with a low risk of severe side effects. Lipidated subunit vaccines are non-replicating and thus may be less susceptible to replication interference than live attenuated vaccines. Our study suggests that the lipidated subunit vaccine approach could be applied to other serotypes of dengue virus as well as other pathogens.

## Introduction

Dengue viruses belong to the *Flavivirus* genus of the *Flaviviridae* family and include four antigenically different serotypes of dengue virus [1]. Dengue virus is a growing threat to public health, not only in terms of geographical distribution but also with respect to infection cases. Dengue occurs in as many as 128 countries throughout tropical and subtropical areas [2]. Vaccination has been proposed as a cost-effective strategy to combat the threat of infectious disease. Unfortunately, an approved dengue vaccine is not presently available, despite tremendous efforts in previous decades. Several vaccine candidates are proceeding in clinical trials [3]. The most advanced candidate is Sanofi Pasteur's recombinant live, attenuated tetravalent dengue-yellow fever chimeric virus vaccine. These vaccine candidates are based on the backbone of 17D yellow fever vaccine strain, each expressing the pre-membrane and envelope genes of one of the four dengue virus serotypes [4]. Recently, the results of a phase 2b trial of this tetravalent dengue vaccine in Thai schoolchildren of 4–11 years of age were reported [5]. The overall efficacy of the vaccine was 30.2%. One or more doses of the vaccine reduced the incidence of dengue-3 and dengue-4 febrile diseases by 80–90%, with a smaller reduction in diseases caused by dengue-1. However, there was no efficacy against dengue-2. Thus, there is an urgent need to complement the deficiency of the recombinant live, attenuated tetravalent dengue-yellow fever chimeric virus vaccine.

In most cases, dengue viral infection causes dengue fever, which is a self-limiting illness. However, infection with dengue virus can also develop into severe dengue hemorrhagic fever (DHF) or dengue shock syndrome (DSS) [6], [7]. The mechanisms of DHF and DSS are still not fully understood. The pathogenesis of DHF and DSS may be due to antibody-dependent enhancement (ADE). ADE is mediated by nonneutralizing antibodies or subneutralizing antibody concentrations bound to the dengue virion, which enhances viral entrance into target cells via the Fc receptor (FcR) [8]. ADE is also mediated by dual-specific antibodies, which bind to both dengue virus particles and target cells lacking FcR expression [9]. In addition to ADE, dengue viral proteins induced antibodies cross-react with plasminogen, endothelial cells, and platelets have been proposed to play an important role in the pathogenesis of DHF and DSS [10]–[12]. The complex pathogenesis of DHF and DSS represents a barrier that complicates dengue vaccine development.

Dengue envelope protein is the major structural protein that mediates dengue virus infection. The envelope protein domain III (ED III) is responsible for viral attachment by binding to the cellular receptor [13], [14]. ED III has been proposed as a suitable target for dengue vaccine development [15]. The immunogenicities of purified recombinant envelope protein or ED III have been evaluated in mice and nonhuman primates [16]–[19]. However, these purified recombinant proteins are poorly immunogenic. Adjuvants are often required in vaccine formulations to augment the immune response to antigens. However, aluminum-containing adjuvants, which are the most widely used in human vaccines, may not be suitable for use in dengue subunit vaccines to induce robust immune responses.

Antigens and immunostimulators are two major components of modern subunit vaccines. We [20] and others [21]–[23] have demonstrated that both bacterial-derived lipoproteins and synthetic lipopeptides can activate antigen-presenting cells via the Toll-like receptor signaling pathway and augment humoral and cellular responses. Based on these findings, we have developed technology to express recombinant lipoprotein in high yields for the development of subunit vaccines with high immunogenicity [24]. In the present study, we prepared recombinant lipidated dengue-2 ED III (LD2ED III) and evaluated its immunogenicity. We demonstrated that exogenous adjuvant is not required for the induction of a robust immune response to LD2ED III. These results provide important information for future clinical studies of ED III-based subunit vaccines.

## Materials and Methods

### Ethics statement

Animal studies were conducted in strict accordance with the recommendations of Taiwan's Animal Protection Act. The protocols were approved by the Animal Committee of the National Health Research Institutes (Protocol No: NHRI-IACUC-098014) and performed according to their guidelines.

### Cloning and expression of recombinant proteins

The amino acid sequence of D2ED III was described previously [25]. Briefly, the sequence of D2ED III was obtained by aligning 13 amino acid sequences from different isolates of type II dengue (accession numbers P07564, P14339, P30026, P27914, Q9WDA6, P12823, P14338, P14337, P14340, P18356, P29984, P29990, and P29991). Based on the amino acid sequence of D2ED III, the DNA sequence of D2ED III using *Escherichia coli* codon usage was determined and fully synthesized by a biotechnology company (Purigo Biotechnology Co., Taipei, Taiwan). The synthesized DNA was then amplified by PCR. To generate an expression plasmid for D2ED III production, the following primers were used: forward primer, 5′-GGAATTCCATATGaaaggcatgagctatgC-3′ (NdeI site, underlined); reverse primer, 5′-CCGCTCGAGgctgctgcctt-3′ (XhoI site, underlined). The PCR product was then cloned into the NdeI and XhoI sites of the expression vector pET-22b(+) (Novagen, Madison, WI) to produce the plasmid pD2DE III. As a result, the C-terminus of the recombinant protein contained a hexahistidine tag (His-tag). To express protein, *E. coli* BL21 (DE3) (Invitrogen, Carlsbad, CA) was transformed with pD2DE III. The transformed cells were cultured at 37°C overnight, and protein expression was induced by adding 1 mM isopropylthiogalactoside (IPTG), followed by incubation for 3 hours at 37°C. To clone and express LD2ED III, the D1 domain and the lipid signal peptide of the lipoprotein Ag473 [24] were cloned into the NdeI and BamHI sites of the expression vector pET-22b(+) (Novagen, Madison, WI) to obtain the plasmid pLipo. The D2ED III gene was cloned into the BamHI and XhoI sites of the pLipo plasmid to produce the plasmid pLD2DE III. As a result, the C-terminus of the recombinant protein contained a His-tag. *E. coli* C43(DE3) (Lucigen, Middleton, WI) was transformed with pLD2DE III to express lipidated protein. The transformed cells were cultured at 37°C overnight. One ml of the overnight culture was scaled up to 600 ml in a 2 l-shake flask and incubated at 37°C for 4 h before induction. Protein expression was induced (OD_600_ = 0.8) by adding 1 mM IPTG, followed by incubation at 20°C for 20 h.

### Production of D2ED III and LD2ED III

D2ED III was purified by disrupting the harvested cells in a French press (Constant Systems, Daventry, UK) at 27 Kpsi in homogenization buffer [20 mM Tris (pH 8.0), 50 mM sucrose, 500 mM NaCl and 10% glycerol]. The cell lysate was clarified by centrifugation (80,000× *g* for 40 min). Most of the D2ED III was present in inclusion bodies. D2ED III was then solubilized with extraction buffer [50 mM NaH_2_PO_4_/5 mM EDTA/200 mM NaCl/0.5 M urea/1% Triton X-100 (pH 6.0)]. The extracted fraction was loaded onto immobilized metal affinity chromatography (IMAC) columns (QIAgen, Hilden, Germany) to purify D2ED III. The eluent from the IMAC column was further purified by passage through an anion exchange column (DEAE Sepharose fast flow; GE) after dialysis against DEAE buffer [50 mM NaH_2_PO_4_/1 M urea (pH 5.8)]. An E membrane (Pall Co., USA) was used to remove endotoxin. The endotoxin levels of the purified D2ED III were determined by the Limulus amebocyte lysate (LAL) assay (Associates of Cape Cod, Inc., Cape Cod, MA), and the resulting endotoxin levels were less than 0.06 EU/mg. After dialysis against 10 mM sodium acetate/3 mg/ml sucrose/4 mM glycine, the D2ED III was lyophilized and stored at −20°C. The fractions from each step were analyzed by SDS-PAGE and immunoblotted with anti-His-tag antibodies.

The disruption and purification steps in the production of LD2ED III were similar to those used for D2ED III. However, LD2ED III was not subjected to anion exchange chromatography. After IMAC purification of LD2ED III, Endotoxin Removing Gel (Pierce, Rockford, IL, USA) was used to remove lipopolysaccharide (LPS). The LPS levels of the purified LD2ED III were determined by LAL assay, and the resulting LPS levels were less than 0.06 EU/mg. After the LD2ED III was dialyzed against 10 mM sodium acetate/3 mg/ml sucrose, the LD2ED III was lyophilized and stored at −20°C. The fractions from each step were analyzed by SDS-PAGE and immunoblotted with anti-His-tag antibodies.

### Identification of the lipid moiety in LD2ED III

LD2ED III was digested with trypsin (Sigma, St. Louis, MO). After digestion, the reaction mixture was further purified with a ZipTip (Millipore, Massachusetts). A 1-µl aliquot of the ZipTip-polished tryptic fragments was mixed with 1 ml of a saturated solution of α-cyano-4-hydroxycinnamic acid in acetonitrile/0.1% trifluoroacetic acid (1∶3 vol∶vol). One microliter of the mixture was placed on the target plate of a MALDI micro MX mass spectrometer (Waters, Manchester, UK) for analysis.

### Virus

Dengue-1/Hawaii, dengue-2/PL046, dengue-3/H-087, and dengue-4/H241 were used for this study. The virus was laboratory-adapted virus and kindly provided by Yi-Ling Lin of the Institute of Biomedical Sciences, Academia Sinica, Taiwan [26], [27]. The virus was propagated in C6/36 cells, and viral titers were determined by focus-forming assays with BHK-21 cells. Briefly, a monolayer of BHK-21 cells in 24-well plates was inoculated with supernatant obtained from C6/36 cultured medium infected with dengue virus. Supernatants were diluted by 10-fold serial dilution (starting at 1∶10). Viral adsorption was allowed to proceed for 3 h at 37°C. An overlay medium containing 2% fetal bovine serum and 0.8% methylcellulose in DMEM was added at the conclusion of adsorption. The infected monolayer was incubated at 37°C. After 72 h of infection, the overlay medium was removed from the wells, and the BHK cells were washed with cold PBS. The cells were fixed for 15 min in 3.7% formaldehyde/PBS. After washing with PBS, the cells were permeabilized with 0.1% Nonidet P-40/PBS for 15 min and blocked with 3% BSA/PBS for 30 min. Infected cells were detected with a monoclonal anti-dengue antibody (American Type Culture Collection, No. HB-114). After washing with PBS, antibody-labeled cells were detected with a secondary antibody conjugated to HRP. The labeling was visualized with 3,3′,5,5′-tetramethylbenzidine (TMB). The focus-forming units (FFUs) were counted, and the viral titers were determined by times dilution factor.

### Mouse experiments

Five BALB/c mice (6–8 weeks of age) were immunized subcutaneously with recombinant D2ED III or LD2ED III. The lyophilized D2ED III and LD2ED III were reconstituted with PBS. Each mouse was received 10 µg/0.2 mL per dose. Mice were given 2 immunizations at a 2-week interval with the same regimen. This immunization protocol was used throughout the present study. Mice were inoculated intraperitoneally with live dengue-2 virus (1×10^7^ FFUs) on the same schedule for comparison with the D2ED III and LD2ED III vaccine candidates. Blood was collected from each mouse at different time points as indicated. Sera were prepared and stored at −20°C until use.

### Measurement of antibody titer

The levels of anti-D2ED III IgG in the serum samples were determined by titrating the samples. Sera were diluted by 3-fold serial dilution (starting at 1∶33). Briefly, purified D2ED III was coated onto 96-well plates. Bound IgG was detected with HRP-conjugated goat anti-mouse IgG Fc. After the addition of TMB, the absorbance was measured with an ELISA reader at 450 nm. ELISA end-point titers were defined as the serum dilution that produced an OD value of 0.5. The serum dilution was obtained from the titration curve by interpolation, unless the OD value was less than 0.5 at the starting dilution (1∶33).

### Measurement of antibody avidity

Antibody avidity was determined on the basis of D2ED III-specific IgG dissociation induced by the chaotropic agent ammonium thiocyanate. Briefly, purified D2ED III was coated onto 96-well plates. After blocking with 1% bovine serum albumin (BSA)/PBS, serum at a dilution of either 1∶100 or 1∶300 was incubated at room temperature for 1 h. The plates were washed and incubated with 0–3.5 M ammonium thiocyanate in 0.5 M increments at room temperature for 15 min. The bound IgG was detected with HRP-conjugated goat anti-mouse IgG. After the addition of TMB, the absorbance at 450 nm was measured with an ELISA reader. The avidity index was calculated as the concentration of ammonium thiocyanate that resulted in a 50% decrease in the initial absorbance [28], [29].

### Focus reduction neutralization tests (FRNT)

Sera were diluted via 2-fold serial dilutions (starting at 1∶8), and the sera were heat-inactivated prior to testing. A monolayer of BHK-21 cells in 24-well plates was inoculated with dengue-2 virus that had been pre-mixed at 4°C overnight with preimmunization or postimmunization sera to a final volume of 0.5 ml. The virus titer prior to pre-mixing was approximately 20–40 FFU per well. The FFUs were obtained as described previously, and the neutralizing antibody titer FRNT_50_ was calculated as the reciprocal of the highest dilution that produced a 50% reduction in FFU compared with control samples containing the virus alone. For calculation purposes, the neutralizing antibody titer was designated as 4 when the neutralizing antibody titer was less than 8.

### Antibody-dependent enhancement tests

Antibody-mediated enhancement of dengue virus infectivity was determined by flow cytometry in K562 cells. Sera were diluted via 4-fold serial dilutions (starting at 1∶8), and the sera were heat-inactivated prior to testing. Serially diluted sera and virus were mixed and incubated to form immune complexes for 1 h at 37°C. K562 cells were mixed with immune complexes (MOI = 0.1) and then incubated for 1.5 h at 37°C. After washing, the cells were resuspended in fresh medium and incubated for 3 days at 37°C. Infections with and without virus were performed in parallel as controls. Cells were stained for intracellular with monoclonal anti-dengue antibodies (American Type Culture Collection, No. HB-114 for dengue-1, dengue-2, and dengue-4; HB-49 for dengue-3). Antibody-labeled cells were detected with a secondary antibody conjugated to FITC. The data were acquired with CellQuest Pro software on a BD FACSCalibur flow cytometer and were analyzed with FACS 3 software. The fold enhancement was defined as the percentage of infected cells in the presence of sera divided by the percentage of infected cells in the absence of sera.

### Statistical analyses

Statistical analyses were performed with the ANOVA Bonferroni post test using GraphPad Prism version 5.02 (GraphPad Software, Inc.). Differences with a *p* value of less than 0.05 were considered statistically significant.

## Results

### Preparation of recombinant antigens containing dengue-2 envelope protein domain III

**Figure 1:**
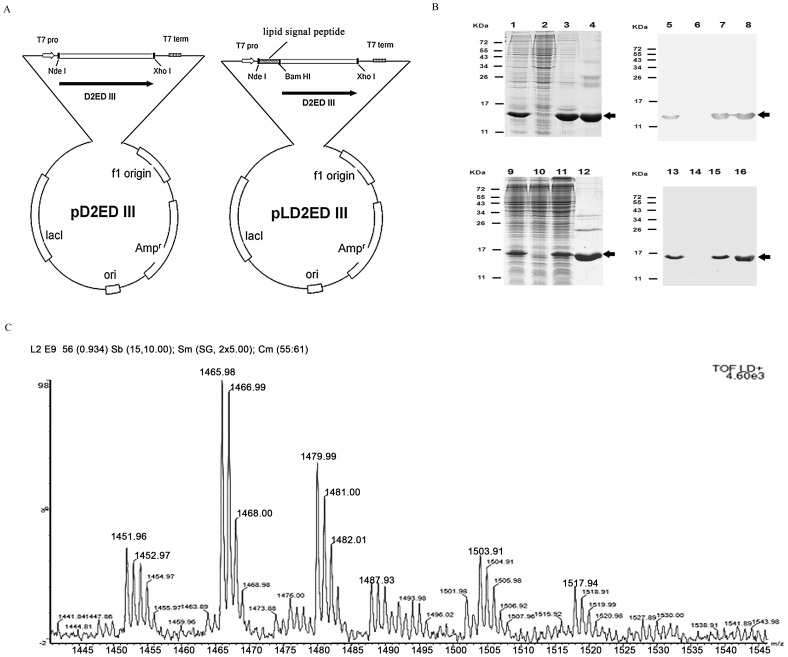
Production and identification of recombinant D2ED III and LD2ED III. (A) The amino acid sequence of D2ED III is a consensus sequence of dengue virus type II (reference [25]). The DNA sequence encoding D2ED III was optimized for *E. coli* codon usage and fully synthesized by a biotechnology company (see Materials and Methods). The PCR product was cloned into a pET22b-based vector to generate the expression plasmid pD2ED III for the production of D2ED III. To clone LD2ED III, pD2ED III was cloned into the pET22b-based vector with a lipid signal peptide in front of the D2ED III gene to generate pLD2ED III for LD2ED III production. Both recombinant proteins contained an additional hexahistidine sequence (His-tag) at their C-termini and were expressed under the control of the T7 promoter. (B) The purification of the D2ED III protein was monitored by 15% reducing SDS-PAGE followed by Coomassie Blue staining and immunoblotting with anti-His-tag antibodies (lanes 1 to 8). D2ED III was expressed in *E. coli* BL21 (DE3). Lane 1, D2ED III expression after IPTG induction; lane 2, protein expression in the absence of IPTG induction; lane 3, extraction of D2ED III from inclusion body; lane 4, purified D2ED III. Lanes 5–8 show immunoblotting to monitor the D2ED III induction and purification processes, and the samples in these lanes are the same as those in lanes 1–4, respectively. The arrows indicate the electrophoretic positions of D2ED III in the gels or blots. LD2ED III purification was monitored by 15% reducing SDS-PAGE followed by Coomassie Blue staining and immunoblotting with anti-His-tag antibodies (lanes 9 to 16). LD2ED III was expressed in *E. coli* C43 (DE3). Lane 9, LD2ED III expression after IPTG induction; lane 10, protein expression in the absence of IPTG induction; lane 11, soluble fraction of LD2ED III; lane 12, purified LD2ED III. Lanes 13–16 show immunoblotting to monitor the LD2ED III induction and purification processes, and the samples in these lanes are the same as those in lanes 9–12, respectively. The arrows indicate the electrophoretic positions of LD2ED III in the gels or blots. (C) N-terminal LD2ED III fragments were obtained and identified after digestion of LD2ED III with trypsin. The digested sample was analyzed with a MALDI-TOF mass spectrometer. The MALDI-TOF MS spectra included three major peaks with m/z values of 1452, 1466, and 1480.

The D2ED III gene was cloned into the expression vector pET-22b(+) to produce the plasmids, pD2ED III and pLD2ED III. They were used for the production of recombinant antigens, D2ED III and lipidated D2ED III (LD2ED III), respectively. Both antigens contained an additional hexahistidine sequence (His-tag) at their C-termini and were expressed under the control of the T7 promoter (Figure 1A).

The purification of D2ED III and LD2ED III were monitored and analyzed by SDS-PAGE and immunoblotting (Figure 1B). After removing LPS, the residual LPS in D2ED III and LD2ED III were less than 0.06 EU/mg. The yields of D2ED III and LD2ED III were 40 mg/l and 8 mg/l, respectively. The immunogenicity and efficacy of endotoxin-free D2ED III and LD2ED III were comparatively analyzed in animal models.

We then measured the exact mass of trypsin-digested N-terminal fragments of LD2ED III. Three major peaks with m/z values of 1452, 1466, and 1480 were identified (Figure 1C). These peaks have been previously identified as a lipidation signature in other lipidated proteins [24]. We confirmed that the peaks of LD2ED III were associated with lipidated cysteine residues and verified that LD2ED III contained an N-acetyl-S-diacyl-glyceryl-cysteine at its N-terminus [30].

### Lipidated dengue-2 envelope protein domain III immunization induces durable neutralizing antibody responses

**Figure 2:**
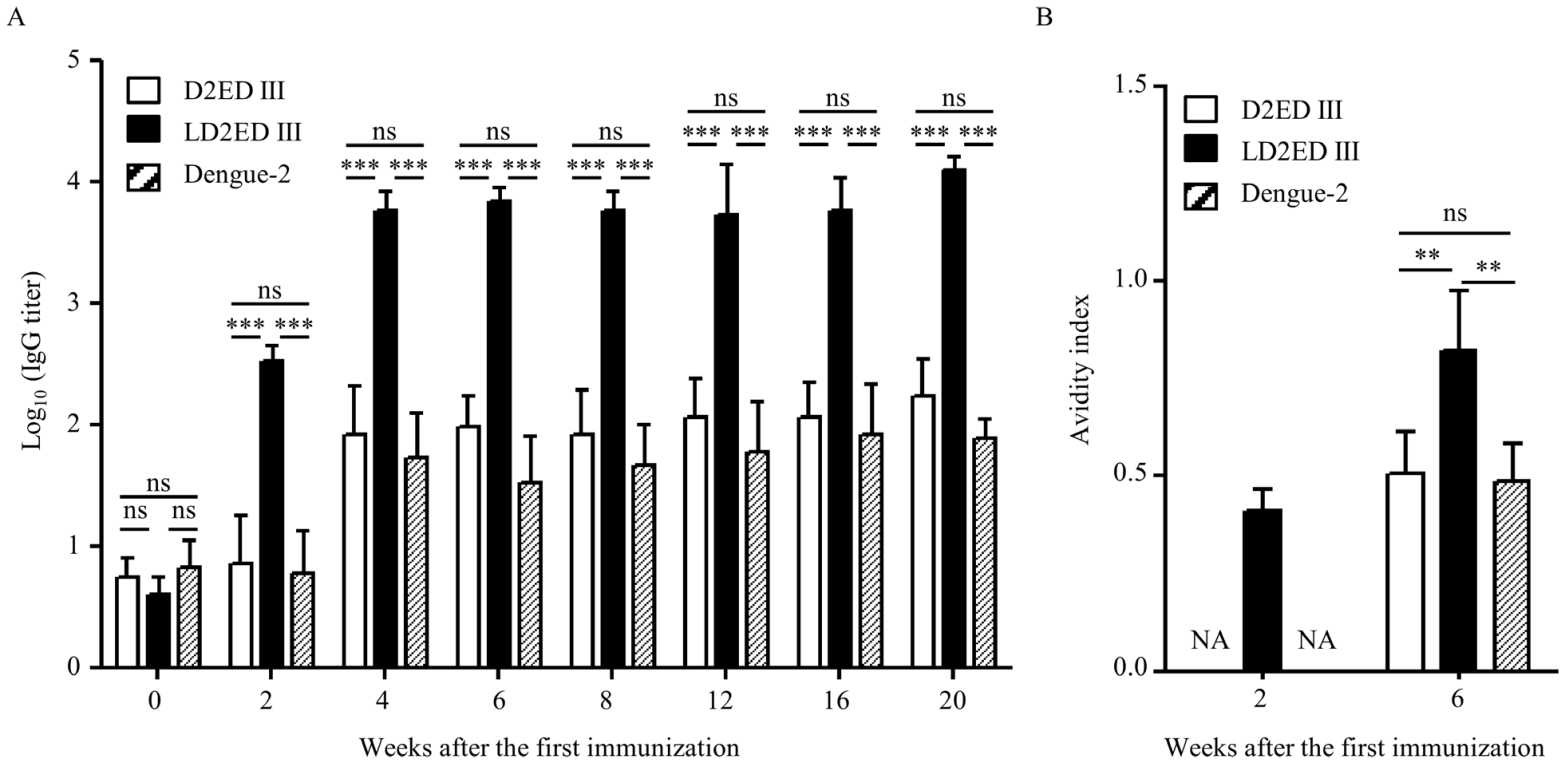
Humoral immune responses induced by vaccine candidates. Groups of BALB/c mice (n = 5) were immunized subcutaneously with 10 µg D2ED III or LD2ED III two times at a two-week interval. Live dengue-2 virus (1×10^7^ FFU) was injected intraperitoneally on the same schedule. Sera were collected from mice at the indicated time points after the first immunization. (A) IgG antibodies against D2ED III were evaluated by ELISA. Preimmune sera (week 0) were collected and used to determine basal levels for comparison. (B) Antibody avidity profiles were examined by ELISA. One of two representative experiments is shown. The results are expressed as the mean ± standard deviation. Statistical significance was determined by the ANOVA Bonferroni post test. ^**^ *p*<0.01; ^***^ *p*<0.001.

The immunogenicity of the purified D2ED III and LD2ED III was tested in mice. Groups of BALB/c mice were immunized with D2ED III or LD2ED III (10 µg per dose) two times with a two-week interval between immunizations. Animals infected with live dengue-2 virus (1×10^7^ FFU) on the same schedule served as controls. Serum samples were collected from the immunized mice at different time points, as indicated in Figure 2. Antibody responses against D2ED III were only detected in D2ED III- or dengue-2 virus-immunized mice after two vaccinations, 4 weeks post-priming (Figure 2A). By contrast, the recombinant LD2ED III was highly immunogenic and induced stronger antibody responses than D2ED III (p<0.05 by the ANOVA Bonferroni post test). Mice immunized with LD2ED III quickly elicited antibody titers 2 weeks post-priming. Antibody titers were further elevated following a booster immunization and were maintained for at least 20 weeks after the initial priming (Figure 2A).

The antibody avidity profiles of serum samples collected from different groups at weeks 2 and 6 were examined. The avidity index was calculated as the concentration of ammonium thiocyanate that resulted in a 50% decrease in the initial absorbance [28], [29]. As shown in Figure 2B, the avidity index of mice immunized with LD2ED III was 0.41±0.06 at 2 weeks after priming. However, mice immunized a single time with D2ED III or dengue-2 virus did not generate significant antibody responses for avidity analysis. After booster immunization, the avidity indexes of mice immunized with D2ED III and dengue-2 virus were 0.50±0.11 and 0.49±0.10 at week 6, respectively. Remarkably, the avidity index of mice immunized with LD2ED III increased to 0.82±0.15 by week 6 and was significantly higher than the avidity index of mice immunized with D2ED III or dengue-2 virus.

**Figure 3:**
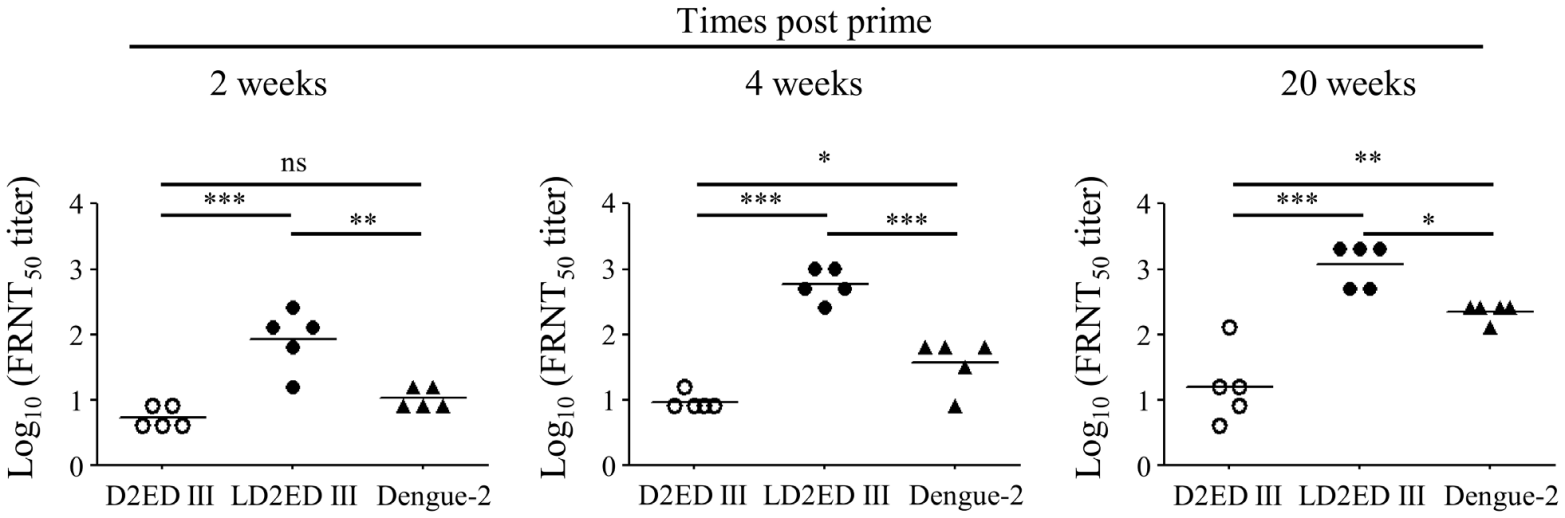
Neutralizing antibody titers in mice immunized with vaccine candidates. Groups of BALB/c mice (n = 5) were immunized subcutaneously with 10 µg D2ED III or LD2ED III two times at a two-week interval. Live dengue-2 virus (1×10^7^ FFU) was injected intraperitoneally on the same schedule. Sera were collected from mice at the indicated time points after the first immunization. The dengue-2 virus neutralizing capacity was determined by FRNT. The neutralizing antibody titer was calculated as the reciprocal of the highest dilution that resulted in a 50% reduction in FFU compared to control samples containing the virus alone. One of two representative experiments is shown. Statistical significance was determined by the ANOVA Bonferroni post test. ^*^ *p*<0.05; ^**^ *p*<0.01; ^***^ *p*<0.001.

Next, we evaluated the neutralizing capacity of the antibodies induced by vaccination. As shown in Figure 3, mice immunized with D2ED III could not stimulate significant neutralizing antibody responses (FRNT_50_ = 5) at 2 weeks after priming. Even after booster immunization, the neutralizing antibody titers were 9 and 16 at 4 and 20 weeks after priming, respectively. Dengue-2 virus-infected mice generated low neutralizing antibody titers (FRNT_50_ = 11) at 2 weeks after primary infection. After secondary infection, neutralizing antibody titers increased and reached 37 and 223 at 4 and 20 weeks after primary infection, respectively. Notably, mice immunized with LD2ED III generated significant neutralizing antibody responses (FRNT_50_ = 84) at 2 weeks after priming. After booster immunization, the neutralizing antibody titers were further elevated to 588 and 1176 at 4 and 20 weeks after priming, respectively. These results suggest that mice immunized with LD2ED III without exogenous adjuvant elicit quick and durable neutralizing antibody responses.

### Reduction of the potential risk of antibody-dependent enhancement of infection

**Figure 4:**
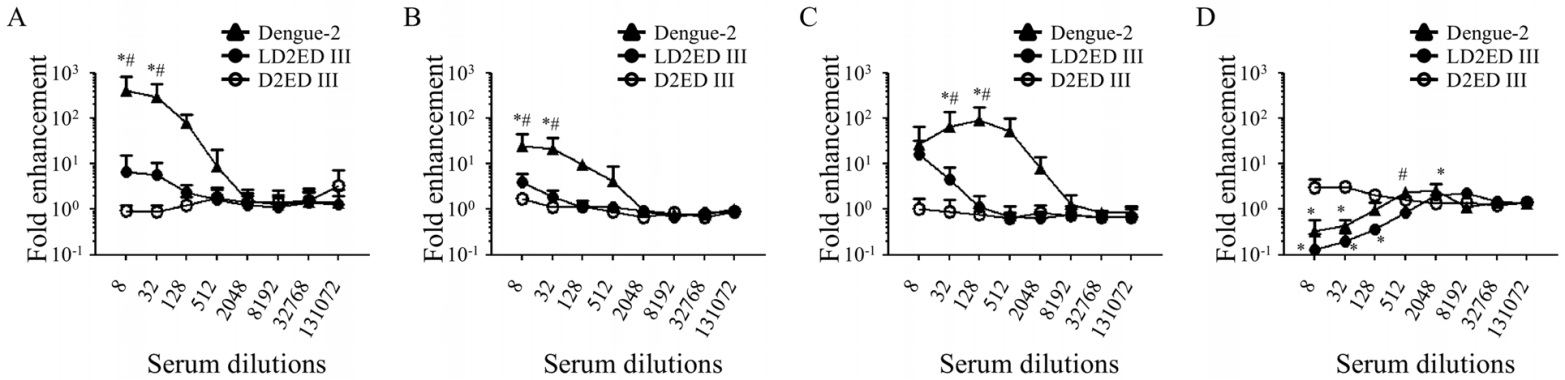
Capacity for antibody-dependent enhancement in mice immunized with vaccine candidates. Groups of BALB/c mice (n = 5) were immunized subcutaneously with 10 µg D2ED III or LD2ED III two times at a two-week interval. Live dengue-2 virus (1×10^7^ FFU) was injected intraperitoneally on the same schedule. Sera were collected from mice at 6 weeks after the first immunization. K562 cells were infected (MOI = 0.1) with (A) dengue-1, (B) dengue-3, (C) dengue-4, or (D) dengue-2 viruses in the presence or absence of serial 4-fold dilutions of pooled serum samples. The fold enhancement values were calculated as the percentage of infected cells in the presence of pooled serum samples divided by the percentage of infected cells in the absence of pooled serum samples. The results are expressed as the mean ± standard deviation from triplicate. Statistical significance was determined by the ANOVA Bonferroni post test at the same dilution. ^*^ *p*<0.05 compared to D2ED III. ^#^ *p*<0.05 compared to LD2ED III.

Antibody-dependent enhancement of infection is a significant concern in the development of vaccines against dengue virus. Therefore, we measured the capacities of vaccine candidates to mediate antibody-dependent enhancement of infection. We employed K562 cells, which have been widely used for the measurement of dengue virus antibody-dependent enhancement of infection. The results are shown in Figure 4. Serum samples obtained from mice immunized with dengue-2 virus possessed tremendous antibody-dependent enhancement capacities for heterotypic viral infection in K562 cells. The peak fold enhancement values were 442.5±389.1, 28.0±19.1, and 93.1±86.4 for dengue-1, dengue-3, and dengue-4, respectively, at the dilution 1/8–1/128. By contrast, antibodies generated from D2ED III-immunized mice did not promote antibody-dependent enhancement of heterotypic viral infection in K562 cells. Serum samples obtained from mice immunized with LD2ED III displayed minor antibody-dependent enhancement of heterotypic viral infection in K562 cells at the lowest dilution tested (1/8). The fold enhancement values were 10.1±7.1, 4.0±2.2, and 16.5±16.7 for dengue-1, dengue-3, and dengue-4, respectively, which were notably lower than the values for the serum samples obtained from mice immunized with dengue-2 virus. Compared with heterotypic viral infection in K562 cells, only low antibody-dependent enhancement capacities were observed for homotypic viral infection in K562 cells. The peak fold enhancement values were 3.1±0.7, 3.4±0.8, and 2.5±0.5 for serum samples obtained from mice immunized with dengue-2 virus, D2ED III, and LD2ED III, respectively. These results suggest that antibodies elicited by LD2ED III have less capacity for antibody-dependent enhancement than antibodies elicited by dengue-2 viral infection.

## Discussion

The development of novel subunit vaccines relies on a limited number of individual components, namely antigens of the specific pathogen under study. Importantly, the selected antigens must elicit protective immunity against the pathogen. To augment the rational design of subunit vaccines, we expressed LD2ED III as a dengue vaccine candidate using an *E. coli*-based expression system (Figure 1). D2ED III of LD2ED III served as the antigenic component, and the lipid moiety of LD2ED III provided a danger signal that activated the immune system to induce an appropriate adaptive immune response. In the present study, we demonstrated that LD2ED III alone, without exogenous adjuvant, elicited higher D2ED III-specific antibody responses than D2ED III or dengue-2 virus (Figure 2A). In addition, the avidity (Figure 2B) and neutralizing capacity (Figure 3) of the antibodies induced by LD2ED III were higher than those elicited by D2ED III or dengue-2 virus. The above properties would be beneficial to a host during dengue virus infection and suggest that LD2ED III could be a potential dengue vaccine candidate.

The role of antibodies in controlling dengue virus infection is complex. Antibodies are thought to mediate both neutralization and enhancement of dengue virus infection [31], [32]. Antibody-dependent enhancement is the leading theory to explain the higher risk of DHF associated with heterologous serotype viral infections [8]. A reduction in the enhancement capacity of antibodies induced by vaccine candidates should increase the safety of dengue vaccines. Anti-ED III antibody titers in D2ED III-immune sera were comparable with those in dengue-2 virus immune sera (Figure 2A). Surprisingly, there was a remarkable capacity for ADE in dengue-2 virus-immune sera but little ADE capacity in D2ED III-immune sera (Figure 4). Anti-ED III antibody titers in LD2ED III-immune sera were also significantly higher than those in dengue-2 virus-immune sera, and the ADE mediated by LD2ED III-immune sera in K562 cells was lower than that mediated by live virus-stimulated antibodies (Figure 4). D2ED III and LD2ED III only induced anti-ED III antibody responses. However, dengue-2 virus induced antibodies against ED III and other viral antigens. These results suggest that anti-dengue virion antibodies other than ED III are the major antibodies that mediate ADE.

Although the exact mechanism of ADE is still not fully understood, it is believed that antibodies against envelope proteins either neutralize or enhance the viral infection, depending on the concentration and affinity of the antibodies [33], [34]. As shown in Figure 4D, enhancement of dengue-2 with sera from mice vaccinated using infectious dengue-2 was observed. The peak fold enhancement was 3.1±0.7 at the dilution 1/512 - 1/2048. Neutralization was observed with antisera at the dilutions 1/8 - 1/32. Notably, a heterotypic enhancing response occurs at a wide range of serum concentrations (Figure 4A–C). These are general phenomena that a homotypic enhancing response are usually restricted to higher serum dilutions due to high neutralizing capacities, while a heterotypic enhancing response occurs at a wide range of serum dilutions because of little heterotypic neutralizing capacities. Similar profiles were observed in sera obtained from LD2ED III immunized mice for homotypic enhancement, the peak fold enhancement was 2.5±0.5 at the dilution 1/2048 - 1/8192 and neutralization was observed with antisera at the dilutions 1/8 - 1/128 (Figure 4D). Most importantly, sera obtained from LD2ED III immunized mice have less enhancement capacities for heterotypic virus than sera from mice vaccinated using infectious dengue-2 (Figure 4A–C). In contrast, D2ED III immunized mice did not elicit significant neutralizing antibodies. The enhancement infection was observed at the sera dilutions 1/8 - 1/32 (Figure 4D). These results suggest that LD2ED III is a good vaccine candidate with low risk of antibody-dependent enhancement. Recently, Mady et al. demonstrated that the delivery of dengue virus to the cell surface at a location other than Fc receptors by a bispecific antibody can also increase viral infectivity [35]. Furthermore, Huang et al. observed that anti-prM antibodies were cross-reactive with heat shock protein 60, which enhanced dengue virion binding and infection of cells lacking Fc receptors [9]. LD2ED III induced high-affinity antibody responses (Figure 2B) while providing only the ED III antigen. These properties could partly explain the notably lower ADE capacity of LD2ED III compared to dengue-2 virus.

Some conserved motifs located in the dengue envelope protein domain II and non-structural protein-1 have been shown to induce autoantibodies. Cross-reaction of dengue viral protein-induced antibodies with host antigens can trigger cell damage or induce harmful effects, which may facilitate DHF/DSS development [9]–[12]. Indeed, such autoantibodies were detected in DHF/DSS patients [10], [36]. All dengue viral antigens are absent from the LD2ED III candidate with the exception of ED III, which may not induce cross-reactive antibodies. Taken together, these results suggest that LD2ED III is a safe vaccine candidate in terms of its reduced ADE capacity and autoantibody induction of LD2ED III.

The results of the current study suggest that the use of lipidated ED III from the four serotypes of dengue virus may have potential for the development of tetravalent dengue vaccines. Alternatively, the strategy of priming with live attenuated dengue vaccines followed by boosting with a lipidated ED III vaccine candidate may enhance ED III-specific immune responses to elicit safe and effective immunity against dengue virus infection. In conclusion, LD2ED III is an effective dengue vaccine candidate for inducing long-lasting neutralizing antibody responses with a low risk of detrimental effects. Future work should examine the suitability of this candidate for clinical use.
